# Sarcopenic obesity in nursing home residents: a multi-center study on diagnostic methods and their association with instrumental activities of daily living

**DOI:** 10.1186/s12877-024-04955-w

**Published:** 2024-05-21

**Authors:** Huiyu Tang, Runjie Li, Ruicen Li, Rongna Lian, Xiaoyan Chen, Wenhua Jiang, Jiaojiao Jiang, Ming Yang

**Affiliations:** 1 Center of Gerontology and Geriatrics, West China Hospital, Sichuan University, Chengdu, China; 2 Health Management Center, West China Hospital, Sichuan University, Chengdu, China; 3 Rehabilitation Center, West China Hospital, Sichuan University, Chengdu, China; 4 West China Hospital, National Clinical Research Center for Geriatrics, Sichuan University, Chengdu, China

**Keywords:** Obese sarcopenia, Functional assessment, Physical function, Long-term care, Muscle quality

## Abstract

**Background:**

Sarcopenic obesity (SO) in nursing home residents is rarely studied. We aimed to evaluate and compare the prevalence and consistency of different SO diagnostic methods and to investigate which criterion demonstrated a stronger association with instrumental activities of daily living (IADL) disability.

**Methods:**

We consecutively recruited older adults aged ≥ 60 years, residing in 15 nursing homes in Zigong City, China. Sarcopenia obesity was defined according to the European Society for Clinical Nutrition and Metabolism (ESPEN) and the European Association for the Study of Obesity criteria (SO_ESPEN_), recommending skeletal muscle mass (SMM) adjusted by body weight (SMM/W) to identify low muscle mass. Further, we adapted ESPEN criteria (SO_ESPEN−M_) by employing SMM adjusted by body mass index (SMM/BMI).

**Results:**

We included 832 participants (median age 73.0 years, 296 women). The prevalence of SO_ESPEN_ and SO_ESPEN−M_ was 43.5% and 45.3%, respectively. SO_ESPEN_ showed good consistency with SO_ESPEN−M_ (Cohen’s kappa = 0.759). More than one-third of participants in the normal weight group were diagnosed with SO_ESPEN_ or SO_ESPEN−M_. Even within the underweight group, the prevalence of SO_ESPEN_ and SO_ESPEN−M_ was 8.9% and 22.2%, respectively. Participants with IADL disability had significantly lower SMM/W and SMM/BMI, but higher fat mass percentage of body weight (FM%) than participants without IADL disability. After full adjustment for potential confounders, SO_ESPEN−M_ (OR 1.68, 95% CI 1.21 to 2.32), but not SO_ESPEN_ (OR 1.28, 95% CI 0.93 to 1.75), remained significantly associated with IADL disability.

**Conclusions:**

Both SO_ESPEN_ and SO_ESPEN−M_ showed a high prevalence among nursing home residents, even among individuals with underweight or normal weight. While SO_ESPEN_ had a good consistency with SO_ESPEN−M_, only SO_ESPEN−M_ was independently associated with IADL disability. Screening and diagnosis of SO should be conducted in nursing home residents irrespective of BMI.

**Supplementary Information:**

The online version contains supplementary material available at 10.1186/s12877-024-04955-w.

## Introduction

Obesity has become a major public health problem around the world. According to the World Obesity Atlas 2023 Report [1], the prevalence of adult obesity is supposed to reach up to 18% by 2035, leading to a huge health and economic burden. Due to the negative impact of fat accumulation, including chronic inflammation, insulin resistance, and oxidative stress, obesity may precipitate the loss of muscle mass and function, known as sarcopenia [2]. The coexistence of sarcopenia and obesity has been proposed as the concept of sarcopenic obesity (SO) [3]. Current literature indicates that their simultaneous presence frequently gives rise to cumulative adverse effects, substantially augmenting the susceptibility to functional disability [4–6].

The prevalence of SO exhibits substantial variation, influenced by differences in study populations, definitions, and cut-off values employed across various studies [7]. The absence of universally recognized diagnostic criteria for SO constitutes a significant impediment to the accurate identification of affected patients and hampers the reliable assessment of prevalence. Therefore, the European Society for Clinical Nutrition and Metabolism (ESPEN) and the European Association for the Study of Obesity (EASO) [8] recently proposed the first international definition and diagnostic criteria for SO, which needs to be validated in different clinical settings. Our team has revealed that the ESPEN/EASO-defined SO (SO_ESPEN_) was an independent prognostic factor for mortality in patients with advanced non-small cell lung cancer [9].

Nursing home residents frequently exhibit a sedentary lifestyle and a propensity for inactivity [10], fostering a detrimental cycle of fat accumulation and muscle depletion [11]. Consequently, it is anticipated that SO would be prevalent in this demographic. Research by Halil et al. [12] indicated that the prevalence of SO reached 22.0% (13.7% in men and 30.2% in women) within nursing homes in Turkey, as identified through low handgrip strength and elevated body mass index (BMI). Similarly, Altinkaynak et al. [13] found that 13.3% of elderly residents with diabetes mellitus in nursing homes were affected by SO, based on the European Working Group on Sarcopenia in Older People (EWGSOP) criteria combined with BMI assessment. However, these studies did not employ the ESPEN/EASO diagnostic criteria for SO, highlighting a gap in the application and validation of these criteria within nursing home populations.

Notably, the ESPEN/EASO group suggests the adjustment of skeletal muscle mass (SMM) for body weight (SMM/W) to determine low muscle mass as a component of SO_ESPEN_ [8]. Given that body size is determined by both weight and height, a more preferable approach for adjusting SMM may involve utilizing BMI, denoted as SMM/BMI [14]. For example, SMM/BMI appeared to be better associated with physical performance and frailty than SMM/W in community-dwelling older adults [15]. Therefore, we employed the SMM/BMI metric to determine low muscle mass, thereby modifying the ESPEN/EASO criteria for SO, designated as SO_ESPEN−M_ thereafter.

This study aimed to investigate the prevalence and consistency of SO_ESPEN_ and SO_ESPEN−M_ among nursing home residents. Furthermore, the study aimed to determine which definition demonstrates a stronger association with instrumental activities of daily living (IADL) disability.

## Methods

### Study design and population

We consecutively recruited residents (aged 60 years and older) living in 15 nursing homes in Zigong City, China, between September 2021 and July 2022. We excluded individuals with any of the following conditions: [1] the presence of any implants (i.e., pacemakers, implantable cardioverter defibrillators, or dental implants); [2] any acute illness (i.e., trauma, acute infection, or fracture); [3] a history of mental disorder, major cognitive impairment, or delirium; [4] a history of skeletal muscle diseases (i.e., myositis, progressive muscular dystrophy, or myasthenia gravis); [5] amputation or recent bone fracture; [6] visible edema; and [7] undergoing surgery within three months prior to the enrolment.

The study was approved by the Biomedical Ethics Review Committee of West China Hospital, Sichuan University (No. 2021 − 965). All participants signed a written informed consent.

### Body composition and muscle strength measurement

Body composition was measured by a trained nurse with a multi-frequency segmental bioimpedance analysis (BIA) device (InBody 770, Biospace, Seoul, Korea). The details regarding the measurement of body composition with the BIA device have been reported previously [9]. SMM and fat mass (FM) were measured using the BIA device. The cut-off values for SMM/W, SMM/BMI, and FM percentage of body weight (FM%) for defining the “low muscle mass” and “obesity” components of SO are presented in Table S1.

Handgrip strength (HGS) was measured using a digital dynamometer (EH101, Xiangshan Inc., Guangdong, China) with participants standing upright, maintaining a feet-shoulder width apart stance, and fully extending the elbow. Each participant underwent three trials with their dominant hand, and the maximum recorded value was used for analysis [16]. The cut-off values for HGS for defining the “low muscle strength” component of SO are shown in Table S1.

### Sarcopenic obesity classification

The classification of sarcopenic obesity in this study is comprehensively presented in Supplementary Table S1, detailing the ESPEN/EASO criteria and the specific cut-off values for SO_ESPEN_ and SO_ESPEN-M_. These thresholds conform to the ESPEN/EASO consensus, meticulously adapted for Asian populations to ensure both cultural and physiological applicability. However, for the crucial parameter of SMM/BMI in our modified criteria, directly applicable cut-offs for Asian populations were unavailable. Therefore, we selected the SMM/BMI cut-offs of <1.017 for men and <0.677 for women, as established by Bahat et al. [17], based on their high specificity (> 80.0%). This selection aims to strike an optimal balance between the precise identification of sarcopenic obesity and the reduction of false positives. Furthermore, to assess the impact of SMM/BMI cut-off points on our principal findings, we conducted a sensitivity analysis by using alternative, higher SMM/BMI thresholds (<1.036 for men and <0.770 for women) for defining SO_ESPEN-M_.

### IADL disability and other measurements

IADL disability was defined as requiring assistance on one or more following item(s): utilizing transportation, shopping, using the telephone, and financial management [18].

Body height was measured using a portable stadiometer to the nearest of 0.5 cm, and body weight was measured using a digital scale to the nearest of 0.1 kg. BMI was calculated as the ratio of weight to the square of height (kg/m²). According to the BMI values [19], participants were categorized into four groups: underweight (BMI < 18.5 kg/m^2^), normal weight (BMI 18.5–23.9 kg/m^2^), overweight (BMI 24.0–27.9 kg/m^2^), and obesity (BMI ≥ 28 kg/m^2^). Additionally, waist circumference (WC) was measured at the midpoint between the last palpable rib and the iliac crest [20]. Hip circumference (HC) was measured at the widest part of the buttocks. The waist-hip ratio was calculated as the ratio of WC to HC.

We also collected the following information via face-to-face interviews: age, gender, education, marital status, smoking, alcohol drinking, and chronic diseases (hypertension, diabetes, pulmonary diseases, coronary heart diseases, stroke, and any type of cancer). Additionally, we assessed three geriatric syndromes: falls, polypharmacy, and cognitive impairments. Falls were defined as any sudden descent from one surface to a lower surface and were assessed by asking the question, ‘Did you fall within the past year?‘. Polypharmacy was defined as the concomitant use of five or more medications [21, 22]. Cognition was assessed using the Clock Drawing Test (CDT), where participants received one point for clock contour, numbers, and hands, respectively [23]. The total score ranged from zero to three points, and participants scoring zero to one were diagnosed with cognitive impairment.

### Statistical analysis

Statistical analyses were conducted using R software version 4.2.3 (R Foundation for Statistical Computing, Vienna, Austria) and Origin 2022 (OriginLab Corporation, Northampton, MA, USA). We used histograms and the Shapiro-Wilk test to explore the distribution of continuous data. All continuous data exhibited a skewed distribution. We presented continuous data as median and interquartile boundary values (p25, p75), while categorical data as frequency and percentage. The prevalence of SO_ESPEN_ and SO_ESPEN−M_ were stratified by age groups and BMI groups.

The differences between groups were tested by the Wilcoxon rank-sum (Mann-Whitney) test for continuous variables, and the Chi-square test for categorical variables. The consistency between SO_ESPEN_ and SO_ESPEN−M_ was evaluated by Cohen’s kappa, with a kappa of > 0.75 indicating good consistency, and a kappa of < 0.40 indicating poor consistency. Pearson’s correlation coefficient (r) was used to assess the association of SMM/W and SMM/BMI with age, as well as the association of weight and BMI with SMM.

Moreover, we used univariate and multivariate logistic regression analysis to investigate the possible association of SO_ESPEN_ and SO_ESPEN−M_ with IADL disability. The results are presented as odds ratios (ORs) with 95% confidence intervals (CIs). Model 1 was adjusted for age and sex, while Model 2 was adjusted for age, sex, education, marital status, falls, and cognitive impairment. A two-sided P-value less than 0.05 was deemed statistically significant. To assess the influence of different SMM/BMI cut-off points on our primary outcomes, a sensitivity analysis was performed utilizing alternative SMM/BMI thresholds (<1.036 for men and <0.770 for women) for delineating SO_ESPEN−M_. This entailed a reevaluation through the multivariate logistic regression models to ensure robustness in our findings.

## Results

### Characteristics of the study population

We included 832 participants. Table 1 summarizes the characteristics of the study population. The age of the population ranged from 60 to 97 years (median age 73.0 years), and 35.6% were women. Age, sex, education, marital status, HGS, FM%, SMM/W, SMM/BMI, falls, cognitive impairment, SO_ESPEN,_ and SO_ESPEN−M_ were associated with IADL disability (Table 1).

**Table 1:** Characteristics of the study population

	All n = 832	Without IADL disability n = 338	IADL disability n = 494	P-value
Demographic				
Age (year)	73.0 (68.0, 82.0)	72.0 (66.0, 80.0)	74.0 (69.0, 83.0)	**<0.001**
Women, n (%)	296 (35.6)	135 (39.9)	161 (32.6)	**0.030**
Education (≤ 6 years), n (%)	629 (75.6)	212 (62.7)	417 (84.4)	**<0.001**
Married, n (%)	215 (25.8)	124 (36.7)	91 (18.4)	**<0.001**
Current smoker, n (%)	272 (32.7)	104 (30.8)	168 (34.0)	0.328
Current drinker, n (%)	190 (22.8)	77 (22.8)	113 (22.9)	0.975
Anthropometric				
BMI (kg/m^2^)	23.7 (21.4, 26.3)	23.7 (21.3, 26.0)	23.7 (21.5, 26.6)	0.328
Waist circumference (cm)	86.0 (80.0, 92.0)	86.0 (80.2, 92.0)	86.0 (79.5, 92.3)	0.754
Hip circumference (cm)	94.0 (89.8, 100.0)	94.30 (90.3, 99.3)	94.00 (89.0, 100.0)	0.237
Waist-hip ratio	0.90 (0.86, 0.95)	0.91 (0.86, 0.94)	0.90 (0.86, 0.95)	0.772
HGS (kg)	21.9 (15.9, 27.1)	23.3 (17.6, 29.1)	20.8 (14.5, 26.3)	**<0.001**
Body composition				
FM% (%)	33.6 (27.6, 38.6)	32.8 (26.9, 37.6)	34.1 (28.0, 39.1)	**0.019**
SMM/W (%)	35.3 (32.3, 38.5)	36.0 (32.8, 39.4)	34.9 (31.9, 38.2)	**0.002**
SMM/BMI	0.83 (0.71, 0.97)	0.85 (0.75, 1.00)	0.81 (0.69, 0.95)	**<0.001**
Geriatric syndromes				
Falls, n (%)	138 (16.6)	42 (12.4)	96 (19.4)	**0.008**
Polypharmacy, n (%)	101 (12.1)	44 (13.0)	57 (11.5)	0.521
Cognitive impairment, n (%)	669 (80.4)	230 (68.0)	439 (88.9)	**<0.001**
Chronic diseases				
Hypertension, n (%)	316 (38.0)	127 (37.6)	189 (38.3)	0.841
Diabetes, n (%)	134 (16.1)	54 (16.0)	80 (16.2)	0.933
Pulmonary diseases, n (%)	108 (13.0)	53 (15.7)	55 (11.1)	0.055
Coronary heart disease, n (%)	62 (7.5)	25 (7.4)	37 (7.5)	0.960
Stroke, n (%)	39 (4.7)	13 (3.8)	26 (5.3)	0.342
Cancer, n (%)	5 (0.6)	4 (1.2)	1 (0.2)	0.180
Sarcopenic obesity				
SO_ESPEN_, n (%)				**<0.001**
No	470 (56.5)	224 (66.3)	246 (49.8)	
Yes	362 (43.5)	114 (33.7)	248 (50.2)	
SO_ESPEN−M_, n (%)				**<0.001**
No	455 (54.7)	229 (67.8)	226 (45.7)	
Yes	377 (45.3)	109 (32.2)	268 (54.3)	

BMI, body mass index; ESPEN, European Society for Clinical Nutrition and Metabolism; FM%, fat mass percentage of body weight; HGS, handgrip strength; IADL, instrumental activities of daily living; SMM, skeletal muscle mass; SO, sarcopenic obesity

The significance of the bold values was p < 0.05

### Prevalence of SO_ESPEN_ and SO_ESPEN−M_

The prevalence of SO_ESPEN_ and SO_ESPEN−M_ was 43.5% (362/832) and 45.3% (377/832), respectively. Among men, 49.1% (263/536) participants were classified as having SO_ESPEN_, 55.4% (297/536) having SO_ESPEN−M_; among women, 33.4% (99/296) participants were classified as having SO_ESPEN_, 27.0% (80/296) having SO_ESPEN−M_. There were 320 (38.5%) participants who met both two SO diagnostic criteria, suggesting a good consistency between SO_ESPEN_ and SO_ESPEN−M_ (Cohen’s kappa = 0.759).

**Fig. 1:**
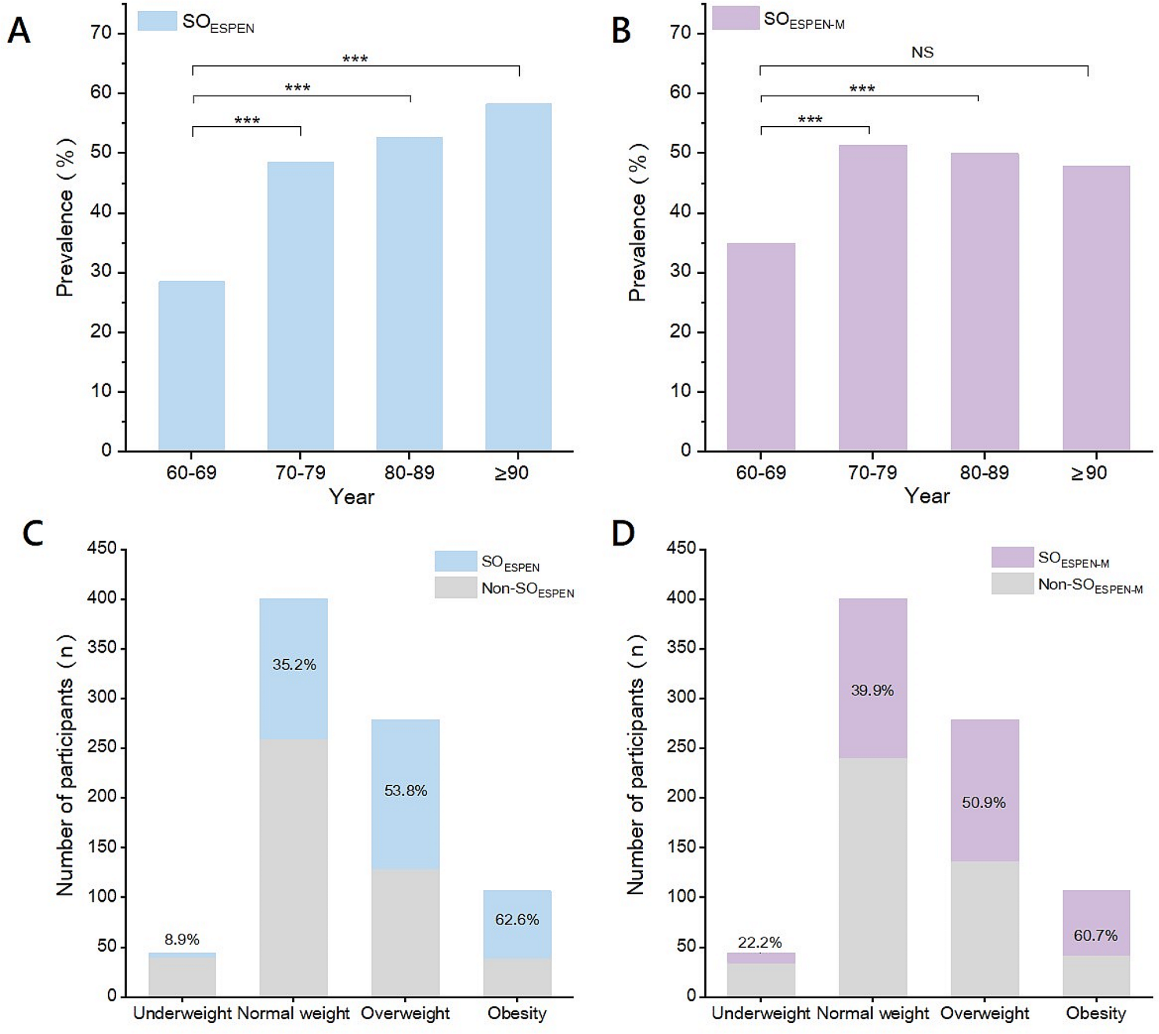
Prevalence of SO_ESPEN_ (**A**) and SO_ESPEN−M_ (**B**) stratified by age groups. Number of participants of SO_ESPEN_ (**C**) and SO_ESPEN−M_ (**D**) stratified by BMI groups. BMI, body mass index; ESPEN, European Society for Clinical Nutrition and Metabolism; SO, sarcopenic obesity. ***P ≤ 0.001; NS, no significance

Figure 1 shows the prevalence of SO_ESPEN_ and SO_ESPEN−M_ stratified by age groups and BMI groups. Not surprisingly, the prevalence of SO_ESPEN_ significantly increased with age group (Fig. 1A). Similar results were observed for SO_ESPEN−M_ (Fig. 1B); however, the prevalence of SO_ESPEN−M_ in the age group over 90 years did not exhibit a statistically significant difference compared to the 60–69 years group (*P* = 0.087).

It is reasonable to observe an increase in the prevalence of either SO_ESPEN_ or SO_ESPEN−M_ with higher BMI (Fig. 1C and D). Notably, more than one-third of the participants in the normal weight group presented with SO_ESPEN_ (35.2%) or SO_ESPEN−M_ (39.9%). Even within the underweight group, the prevalence of SO_ESPEN_ and SO_ESPEN−M_ was 8.9% and 22.2%, respectively.

### Correlations of age, weight, and BMI with muscle mass indicators

**Fig. 2:**
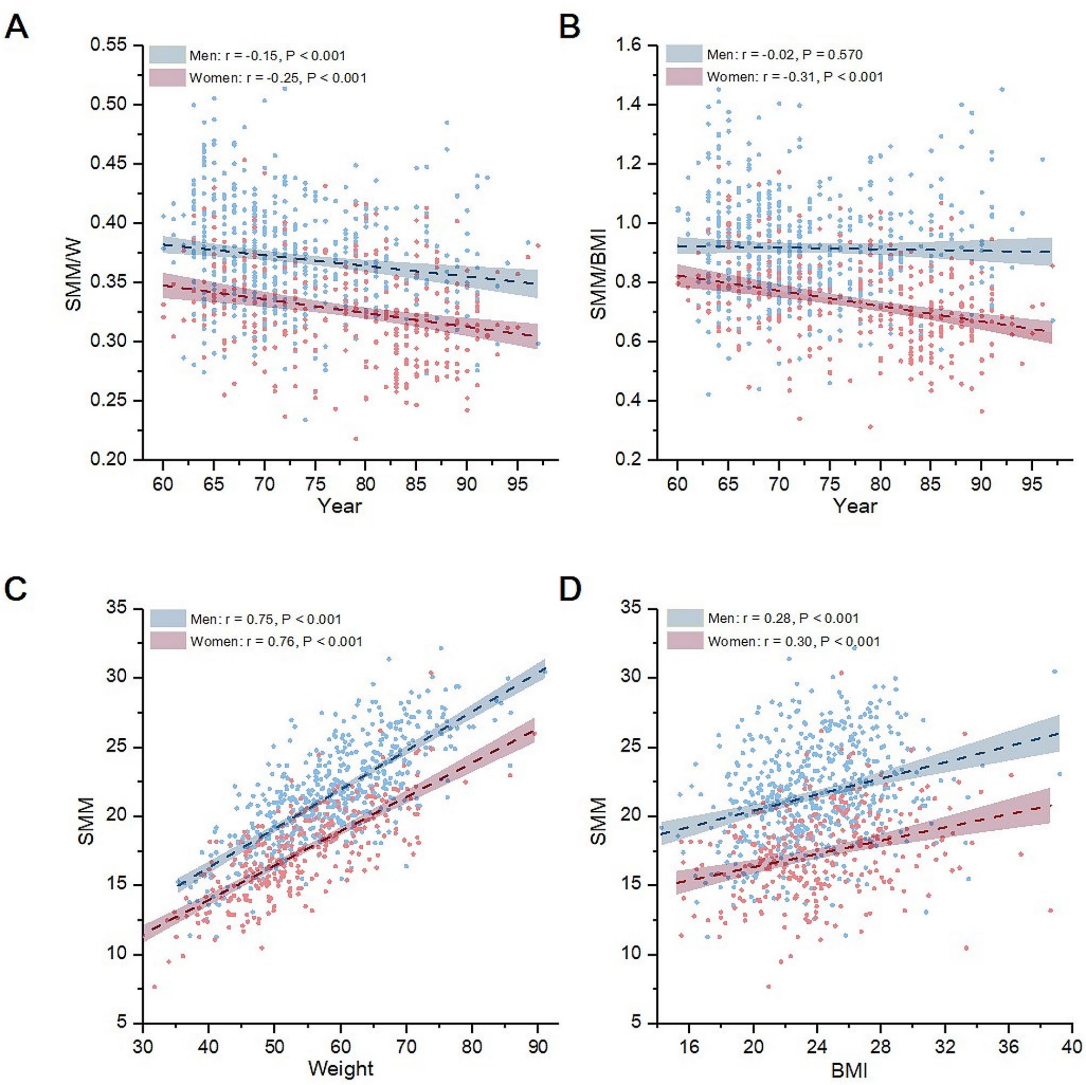
Correlations of SMM/W (**A**) and SMM/BMI (**B**) with age among men and women and correlations of SMM with weight (**C**) and BMI (**D**) among men and women. BMI, body mass index; SMM, skeletal muscle mass

Figure 2 presents scatter plots illustrating the correlations of age, weight, and BMI with muscle mass indicators, stratified by sex. Both SMM/W (*r*=-0.25, *P* < 0.001, Fig. 2A) and SMM/BMI (*r*=-0.31, *P* < 0.001, Fig. 2B) were negatively and slightly correlated with age among women. Among men, SMM/W (*r*=-0.15, *P* < 0.001, Fig. 2A) but not SMM/BMI (*r*=-0.02, *P* = 0.570, Fig. 2B) was correlated with age.

Additionally, among both men and women, SMM exhibited a positive and robust correlation with body weight (men: *r* = 0.75, women: *r* = 0.76, both *P* < 0.001). It also demonstrated a positive albeit slight correlation with BMI (men: *r* = 0.28, women: *r* = 0.30, both *P* < 0.001) (Fig. 2A and B).

### Associations of body composition indicators with IADL disability

**Fig. 3:**
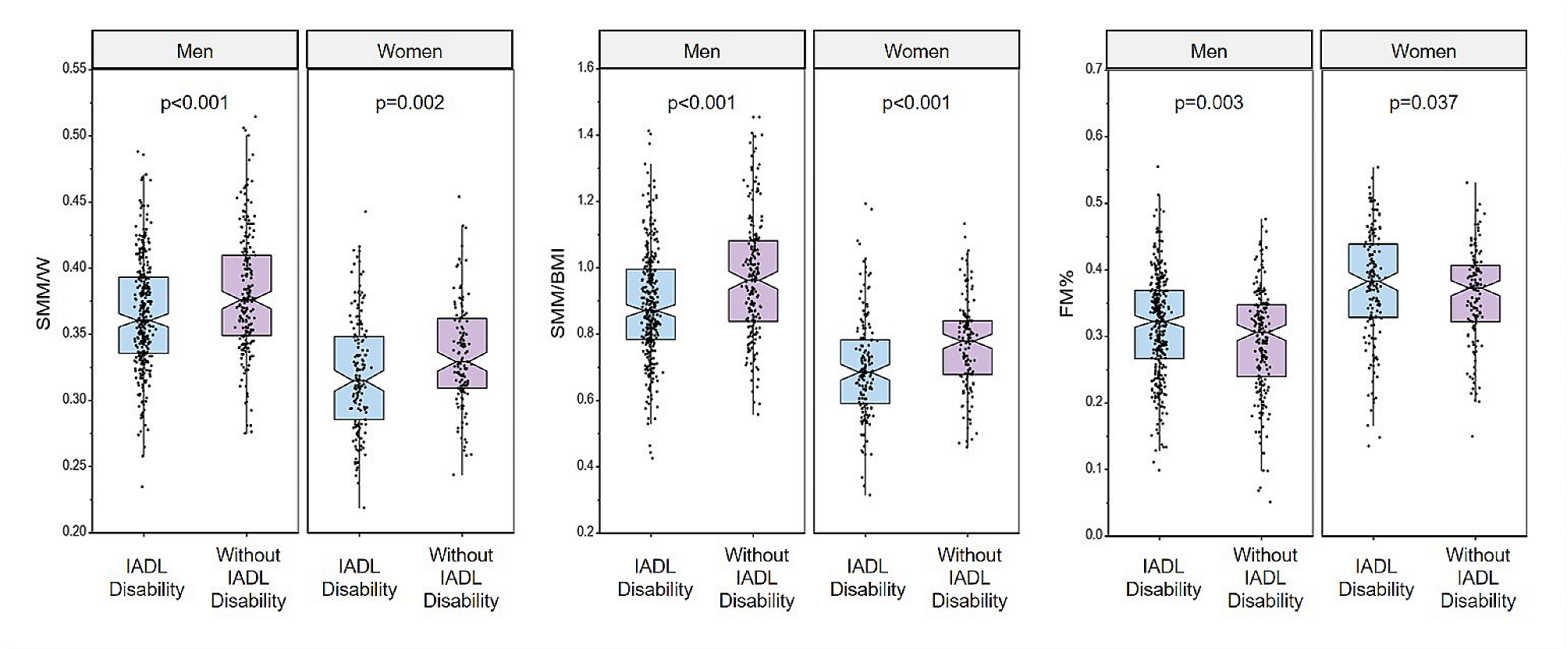
Group comparisons of SMM/W, SMM/BMI, and FM% stratified by sex. BMI, body mass index; FM%, fat mass percentage of body weight; IADL, instrumental activities of daily living; SMM, skeletal muscle mass

As shown in Fig. 3, among both men and women, participants with IADL disability exhibited significantly lower SMM/W (men: *P* < 0.001, women: *P* = 0.002) and SMM/BMI (both *P* < 0.001), but higher FM% (men: *P* = 0.003, women: *P* = 0.037) compared to participants without IADL disability.

### Associations of SO_ESPEN_ and SO_ESPEN−M_ with IADL disability

As presented in Table 2, following adjustment for age and sex, both SO_ESPEN_ (OR 1.66, 95% CI 1.23 to 2.24) and SO_ESPEN−M_ (OR 2.19, 95% CI 1.61 to 2.97) exhibited a significant association with IADL disability. However, with full adjustment, only SO_ESPEN−M_ (OR 1.68, 95% CI 1.21 to 2.32), and not SO_ESPEN_ (OR 1.28, 95% CI 0.93 to 1.75), remained significantly associated with IADL disability. This pattern persisted even when applying alternative SMM/BMI cut-offs, as detailed in Table S2.

**Table 2:** Univariate and multivariate logistic regression models for IADL disability

Characteristic	Univariate Analysis		Multivariate Analysis (Model 1)		Multivariate Analysis (Model 2)	
	OR (95% CI)	P-value	OR (95% CI)	P-value	OR (95% CI)	P-value
SO_ESPEN_						
No	Ref		Ref		Ref	
Yes	1.98 (1.48, 2.63)	**<0.001**	1.66 (1.23, 2.24)	**0.001**	1.28 (0.93, 1.75)	0.131
SO_ESPEN−M_						
No	Ref		Ref		Ref	
Yes	2.49 (1.86, 3.32)	**<0.001**	2.19 (1.61, 2.97)	**<0.001**	1.68 (1.21, 2.32)	**0.002**

ESPEN, European Society for Clinical Nutrition and Metabolism; IADL, instrumental activities of daily living; OR, odds ratio; SO, sarcopenic obesity

Model 1: adjusted for age and sex

Model 2: adjusted for age, sex, education, marital status, falls, and cognitive impairment

The significance of the bold values was p < 0.05

## Discussion

Our study validated the ESPEN/EASO criteria for SO in a multi-center cohort of nursing home residents. Both SO_ESPEN_ and SO_ESPEN−M_ were highly prevalent, exceeding 40% in our study population, even among participants with underweight or normal weight. The prevalence of SO_ESPEN_ and SO_ESPEN−M_ increased with age. SMM exhibited a stronger correlation with body weight than BMI. While both SMM/W and SMM/BMI were associated with IADL disability, only SO_ESPEN−M_ showed a significant association with IADL disability after full adjustment for potential confounders.

After the release of the ESPEN/EASO consensus, some studies have embraced this definition and diagnostic criteria across various study populations, including stoke [24], rehabilitation [25], post-bariatric surgery [26], cancer [9], and community-dwelling older adults [5, 27]. The reported prevalence of SO_ESPEN_ in these studies ranged from 4.3 to 31.9%, whereas it reached 43.5% in our study population. Discrepancies in prevalence among these studies may be attributed to variations in reference populations, diagnostic methods for body composition, and cut-off points for the components of SO.

Existing evidence indicated that the optimal adjustment for SMM when defining the sarcopenia component of SO remains inconclusive [8, 28]. While the ESPEN/EASO criteria recommended using SMM/W to determine low muscle mass, this metric was considered inadequate to account for the body size of people with obesity [29]. Addressing this concern, Bahat et al. [14] proposed using the use of SMM/BMI. Hence, we further modified the ESPEN/EASO-defined SO by employing SMM/BMI to identify low muscle mass, termed SO_ESPEN−M_ in this study. In this study, the prevalence of SO_ESPEN−M_ (45.3%) was found to be comparable to that of SO_ESPEN_ (43.5%). The consistency between the two diagnostic criteria demonstrated good agreement (Cohen’s kappa = 0.759). This finding aligns with our prior study, indicating excellent agreement between SO_ESPEN_ and SO_ESPEN−M_ in patients with non-small cell lung cancer [9].

The prevalence of SO increased with aging, reaching 48.0% and 27.5% in men and women in those aged over 80 years [30]. Interestingly, we found that the prevalence of SO_ESPEN−M_ in the age group over 90 years did not exhibit a statistically significant difference compared to the 60–69 years group. This could be attributed to survivorship bias. Specifically, those who live into their 90s likely constitute a distinct subgroup with unique health and functional profiles, diverging from their younger peers. This suggests a potential leveling off in the risk factors for sarcopenic obesity within this extremely aged cohort. Additionally, physiological and compositional changes associated with aging, such as vertebral compression leading to decreased stature and the reconfiguration of muscle and fat distribution, might impact the assessment of SMM and BMI. Moreover, the limited number of participants over 90 could further contribute to the lack of statistical significance observed. However, existing research has not yet specifically applied the SO_ESPEN−M_ criteria to populations over the age of 90 years. Consequently, additional studies are essential to validate our conclusions in this unique population.

It is noteworthy that a substantial proportion of the study population categorized as underweight and normal weight based on BMI criteria were diagnosed with either SO_ESPEN_ or SO_ESPEN−M_. This finding holds significance as it underscores the necessity of screening SO regardless of BMI. Despite the widespread use of BMI worldwide, it is acknowledged to be an imperfect indicator of obesity [31]. BMI alone is insufficient for assessing FM%, fat adiposity distribution, or the extent of metabolic disturbance [32]. Age-related alterations in body composition, including body fat increases and muscle mass declines [3], can result in minimal changes in total body weight and BMI. For example, Molino et al. [33] found that fat accumulation and redistribution associated with muscle loss did not necessarily lead to an increase in BMI. Hence, neglecting the screening and diagnosis of SO in underweight or normal-weight populations could impede efforts in the prevention and management of SO.

Muscle and bone mass decline with aging, increasing the risk of sarcopenia in later life. Attentionally, our study observed gender differences in SMM/BMI with aging. We found that SMM/BMI was negatively correlated with age in women, but not in men. This is mainly related to the different patterns of body composition changes with aging between men and women. Kin et al. [34] reported that leg lean mass, appendicular lean mass (ALM), and total hip bone mineral density (BMD) showed consistent and slowly progressive decline with aging in men, while presenting accelerated abruptly decline from the age of 75 years in women. Moreover, the roles of sex hormones on muscle biology and bone metabolism are different between men and women. For example, testosterone can increase muscle and bone mass, and decrease inflammatory [3]. On the contrary, the rapid decrease of estradiol at menopause accelerates the decline of muscle mass, and bone mass in postmenopausal women [35]. Therefore, this gender-specific difference might be explained by the gender-related changes on muscle and bone.

Some studies have investigated the association of SMM/W or SMM/BMI with various outcomes. For example, Bahat et al. [15] reported that SMM/BMI, compared to SMM/W, exhibited a stronger association with functional disability in community-dwelling older adults aged ≥ 60 years. Additionally, our prior study demonstrated that SMM/BMI was a better predictor of mortality than SMM/W in patients with non-small cell lung cancer [9]. In the present study, both SMM/W and SMM/BMI were significantly lower in participants with IADL disability compared to participants without IADL disability. Further longitudinal investigations are warranted to comprehensively compare the predictive value of SMM/W or SMM/BMI for various health outcomes. This is essential to determine which metrics would be better for defining low muscle mass.

Our study revealed a significant association between SO_ESPEN-M_ and IADL disability, an association that persisted even after comprehensive adjustments for a range of potential confounders. Notably, this link was not observed with SO_ESPEN_. Crucially, the robustness of our findings is underscored by the consistency observed across different diagnostic thresholds for SMM/BMI, as detailed in Table S2. This consistency across varying cut-offs reinforces the stability and reliability of our results, suggesting that the associations we have identified are not artefacts of particular diagnostic criteria but reflect a genuine relationship within the data. This implies that using SMM/BMI might be more appropriate in identifying the sarcopenia component of SO in our study population.

Previous studies have suggested that sarcopenic obesity may play a role in the development of IADL disability in older adults [36]. For example, a study of 451 elderly men and women followed for up to eight years found that subjects with SO at baseline were two to three times more likely to report the onset of IADL disability during follow-up than those with normal body composition [37]. Another study on community-dwelling older men also found that SO was associated with poor functional outcomes, including IADL disability, independent of confounders [38]. Notably, these studies did not define SO according to the ESPEN/EASO criteria. Recently, Shimizu et al. [25] found that SO_ESPEN_ in patients undergoing rehabilitation was not associated with poor functional outcomes. Therefore, further research is needed to clarify the relationship between different definitions of SO and functional outcomes.

Our study has some limitations. Firstly, due to its cross-sectional design, establishing a cause-effect relationship between SO and IADL disability is not possible. Therefore, further prospective studies are required to validate our findings. Secondly, our study population was exclusively drawn from nursing home residents, warranting caution in generalizing our results to other populations. Thirdly, the measurement of body composition in our study relied on a multi-frequency segmental BIA device. Despite the exclusion of patients with visible edema, it is essential to acknowledge that BIA results may be influenced by the body’s hydration status, including dehydration and latent edema.

## Conclusions

Both SO_ESPEN_ and SO_ESPEN−M_ exhibited high prevalence among nursing home residents. While SO_ESPEN_ had a good consistency with SO_ESPEN−M_, only SO_ESPEN−M_ was independently associated with IADL disability in our study population. However, the question of whether SMM/BMI is better than SMM/W for defining the muscle mass component of SO remains uncertain. Further studies are warranted to investigate this aspect.

Furthermore, a considerable proportion of the study population, classified as underweight and normal weight based on BMI criteria, received diagnoses of either SO_ESPEN_ or SO_ESPEN−M_. Hence, the screening and diagnosis of SO should be conducted in nursing home residents irrespective of BMI.

### Electronic supplementary material

Below is the link to the electronic supplementary material.

Supplementary Material 1

Supplementary Material 2

## Data Availability

The raw data used in this article can be obtained from the corresponding author on reasonable request.

## Abbreviations

ALM: Appendicular Lean Mass
BIA: Bioimpedance Analysis
BMD: Bone Mineral Density
BMI: Body Mass Index
CIs: Confidence Intervals
EASO: the European Association for the Study of Obesity
ESPEN: the European Society for Clinical Nutrition and Metabolism
FM%: Fat Mass Percentage of Body Weight
HC: Hip Circumference
HGS: Handgrip Strength
IADL: Instrumental Activities of Daily Living
ORs: Odds Ratios
SMM: Skeletal Muscle Mass
SMM/BMI: SMM adjusted by Body Mass Index
SMM/W: SMM adjusted by Body Weight
SO: Sarcopenic Obesity
WC: Waist Circumference
