# Supplementary material for: Sarcopenic obesity in nursing home residents: a multi-center study on diagnostic methods and their association with instrumental activities of daily living
Source: BMC Geriatr. 2024 May 21;24:446. doi: 10.1186/s12877-024-04955-w (PMC11110436; doi:10.1186/s12877-024-04955-w)
Supplement: Supplementary file 1 — Supplementary Material 1 [file 12877_2024_4955_MOESM1_ESM.docx]

| **Table S1.** The classifications of SO and corresponding cut-off values | | | | | |
| --- | --- | --- | --- | --- | --- |
|  | Low HGS |  | Low muscle mass |  | Obesity |
| SO_ESPEN_ | Men:＜28kg,  Women:＜18kg (1) |  | Men: SMM/W＜38.2%,  Women: SMM/W＜32.2% (2) |  | Men: FM%＞20.21%,  Women: FM%＞31.71% (3) |
| SO_ESPEN-M_ | Men:＜28kg,  Women:＜18kg (1) |  | Men: SMM/BMI＜1.017,  Women: SMM/BMI＜0.677 (4) |  | Men: FM%＞20.21%,  Women: FM%＞31.71% (3) |

BMI, body mass index; ESPEN, European Society for Clinical Nutrition and Metabolism; FM%, fat mass percentage of body weight; HGS, handgrip strength; SMM, skeletal muscle mass; SO, sarcopenic obesity.

# REFERENCES

1. Chen LK, Woo J, Assantachai P, Auyeung TW, Chou MY, Iijima K, et al. Asian Working Group for Sarcopenia: 2019 Consensus Update on Sarcopenia Diagnosis and Treatment. J Am Med Dir Assoc. 2020;21(3):300-7.e2.

2. Lee J, Hong YP, Shin HJ, Lee W. Associations of Sarcopenia and Sarcopenic Obesity With Metabolic Syndrome Considering Both Muscle Mass and Muscle Strength. J Prev Med Public Health. 2016;49(1):35-44.

3. Kim TN, Yang SJ, Yoo HJ, Lim KI, Kang HJ, Song W, et al. Prevalence of sarcopenia and sarcopenic obesity in Korean adults: the Korean sarcopenic obesity study. Int J Obes (Lond). 2009;33(8):885-92.

4. Bahat G, Kilic C, Ilhan B, Karan MA, Cruz-Jentoft A. Association of different bioimpedanciometry estimations of muscle mass with functional measures. Geriatr Gerontol Int. 2019;19(7):593-7.
