# Supplementary material for: Sarcopenic obesity in nursing home residents: a multi-center study on diagnostic methods and their association with instrumental activities of daily living
Source: BMC Geriatr. 2024 May 21;24:446. doi: 10.1186/s12877-024-04955-w (PMC11110436; doi:10.1186/s12877-024-04955-w)
Supplement: Supplementary file 2 — Supplementary Material 2 [file 12877_2024_4955_MOESM2_ESM.docx]

| **Table S2. Univariate and multivariate logistic regression models for IADL disability** | | | | | | | | |
| --- | --- | --- | --- | --- | --- | --- | --- | --- |
| Characteristic | Univariate Analysis | |  | Multivariate Analysis (Model 1) | |  | Multivariate Analysis (Model 2) | |
|  | OR (95% CI) | P-value |  | OR (95% CI) | P-value |  | OR (95% CI) | P-value |
| SO_ESPEN-M*_ |  |  |  |  |  |  |  |  |
| No | Ref |  |  | Ref |  |  | Ref |  |
| Yes | 2.59 (1.94, 3.44) | **＜0.001** |  | 2.23 (1.65, 3.00) | **＜0.001** |  | 1.73 (1.25, 2.37) | **0.001** |

ESPEN, European Society for Clinical Nutrition and Metabolism; IADL, instrumental activities of daily living; OR, odds ratio; SO, sarcopenic obesity. *The cut-offs for SMM/BMI were＜1.036/0.770 in men and women, respectively.

Model 1: adjusted for age and sex.

Model 2: adjusted for age, sex, education, marital status, falls, and cognitive impairment.
